# Rapid detection of a novel B1-β-lactamase gene, *blaAFM-1* using a loop-mediated isothermal amplification (LAMP) assay

**DOI:** 10.1186/s12941-021-00486-z

**Published:** 2021-12-07

**Authors:** Yingcheng Qin, Xiaonv Duan, Yuan Peng, Yongyu Rui

**Affiliations:** Laboratory Medicine Center, Nanfang Hospital, Southern Medical University, Guangzhou, 510515 China

**Keywords:** Carbapenemase-producing organisms, *BlaAFM-1* gene, B1 subclass metallo-β-lactamase, LAMP

## Abstract

**Background:**

*BlaAFM-1* (GenBank Accession No. 143105.1) is a new B1 subclass metallo-β-lactamase gene discovered by our group, and isolated from an *Alcaligenes faecalis* plasmid that renders carbapenem antibiotics ineffective. In this study, we generated a fast and reliable assay for *blaAFM-1* detection.

**Methods:**

We designed optimum loop-mediated isothermal amplification (LAMP) primers and constructed a recombinant plasmid AFM-1 to specifically detect *blaAFM-1*. Optimal LAMP primers were used to assess sensitivity of the recombinant plasmid AFM-1 and *blaAFM-1*-supplemented samples (simulated sputum and simulated feces). Fifty two samples, without *blaAFM-1*, were used to assess LAMP real-time assay specificity; these samples were verified by conventional PCR and sequencing for the absence of *blaAFM-1.* Three hundred clinical Gram-negative carbapenem-resistant strains were tested by LAMP assay for strains carrying *blaAFM-1*, which were confirmed by conventional PCR and Sanger sequencing. We calculated the sensitivity and its 95% confidence interval (95% CI), specificity and its 95% CI, and predictive values of the LAMP assay and conventional PCR/sequencing by investigating positive and negative clinical strains.

**Results:**

The lowest limit of detection for the recombinant plasmid AFM-1 and *blaAFM-1*-supplemented samples (in both simulated sputum and simulated feces) was 10^1^ copies/reaction. All amplification curves of the 52 *blaAFM-1*-free bacteria strains were negative, suggesting the LAMP assay had excellent specificity for detecting *blaAFM-1*. Among the 300 clinical strains, eight were positive for *blaAFM-1* using LAMP. These LAMP results were consistent with conventional PCR and Sanger sequencing data. As with conventional PCR/sequencing, the LAMP method exhibits 100% sensitivity (95% CI 59.8–100%) and 100% specificity (95% CI 98.4–100%) for *blaAFM-1* detection. The LAMP assay is also time-efficient (1 h) for *blaAFM-1* detection.

**Conclusions:**

We established a new LAMP assay with high sensitivity and specificity to detect the novel B1-β-lactamase gene, *blaAFM-1*.

**Supplementary Information:**

The online version contains supplementary material available at 10.1186/s12941-021-00486-z.

## Background

In recent years, the rate of carbapenem resistance in Gram-negative bacteria has been increasing and is now a worldwide problem [1, 2]. When compared with bacteria susceptible to carbapenem antibiotics, their disease causing potential leads to extremely high morbidity and mortality rates [3], with limited therapeutic options for carbapenem resistance Gram-negative organisms [4].

Carbapenemase production is an important mechanism underpinning bacterial resistance to carbapenem antibiotics. Carbapenemases are divided into three categories; A, B, and D Ambler classes [5, 6]. Ambler class B carbapenemases are also called metallo-β-lactamases (MBLs). Members of the MBLs include NDM (New Delhi metallo-β-lactamase), VIM (Verona integron-encoded metallo-β-lactamase), and IMP (imipenemase metallo-β-lactamase) which have now spread globally [4, 7]. Some bacteria can acquire drug-resistance genes from other bacteria via plasmid conjugation; this process has considerably compromised many clinical settings [4, 8]. Thus, carbapenemase gene detection is of great clinical significance.

In this study, a novel B1-β-lactamase gene *blaAFM-1* (GenBank Accession No. 143105.1) was identified by our group on a plasmid from *Alcaligenes faecalis*. When compared with nucleotide and amino acid homologies of other B1-β-lactamase genes, the highest comparative identities were 85% and 87%, respectively. Conjugation assays indicated that *blaAFM-1* is transferred from the donor strain (*A. faecalis*) to the recipient strain (*Escherichia coli*). Moreover, the Carba NP test also showed that carbapenemase AFM-1 could hydrolyze carbapenems. More interestingly, several strains, i.e., *Comamonas testosteroni, Stenotrophomonas maltophilia, Bordetella trematum,* and *Comamonas aquatica* were shown to harbor *blaAFM-1* in clinical carbapenem-resistant Gram-negative strains.

Loop-mediated isothermal amplification (LAMP) uses six specific primer sets for six different regions of a target sequence. The Bst polymerase, with strand displacement activity and nucleic acid amplification functions, is used to perform nucleic acid amplifications under constant temperature conditions [9–11]. In this study, we established and evaluated a fast and reliable assay for detecting *blaAFM-1* using the LAMP assay.

## Materials and methods

### Primer design

The *blaAFM-1* sequence was downloaded from GenBank (GenBank Accession No. 143105.1). To determine the optimal primer set, several sets of primers were designed by Primer Explorer V4 software (http://primerexplorer.jp/e/). Each set consisted of six primer sequences, one pair of inner primers (BIP and FIP), one outer pair (F3 and B3), and one pair of loop primers (LF and LB). We selected the optimal LAMP primer set, with the highest amplification efficiency. Primers were designed by Guangzhou Bangce Biotechnology Co., Ltd (Guangzhou, China), and synthesized by Sangon Biotech Co., Ltd (Shanghai, China) (Table 1).

**Table 1:** Optimal LAMP primer sets for *blaAFM-1*

Primer	Sequences (5′–3′)	Position^&^ (bp)
F3	TTGGTGAGCAGGTGGATA	95–112
B3	AAGGTCAGGCTGTGCT	479–494
FIP(F1c + F2)	TCATCGGTCCAGGCGGTAGGCAACATACCTCGTTCAT	–
BIP(B1c + B2)	TCAGACCAGCCAGATCCTCAACCATCTTGTCCTGATGCG	–
LF	CGCCATCCTTGACGATCA	241–258
LB	CTGGATTAAGCAAGAGATCAATCTG	300–324

^&^The complete coding sequence of AFM-1 is taken as reference sequences

F3 and B3, outer primers; FIP and BIP, inner primers; LF and BF, loop primers

### Construction of the recombinant plasmid AFM-1

*BlaAFM-1* was chemically synthesized and ligated into the pET28-a(+) vector (Beijing Liuhe Huada Gene Technology Co., Ltd, Beijing, China) to construct the recombinant plasmid AFM-1 (Beijing Liuhe Huada Gene Technology Co., Ltd). Next, the plasmid was transformed into competent *E. coli* Top10 cells (Beijing Liuhe Huada Gene Technology Co., Ltd), and plated onto Luria Broth agar containing 4 μg/mL meropenem for overnight incubation at 37 °C. The next day, plasmid DNA was extracted from colonies using the Qiagen bacterial plasmid extraction kit (TianKangxin (Beijing) Technology Co., Ltd. Beijing, China), and the primers pET-F/R (Sangon Biotech (Shanghai) Co., Ltd) (Table 2) were used to PCR amplify the recombinant plasmid AFM-1. The plasmid was sequenced to verify the correct *blaAFM-1* insert.

**Table 2:** Conventional PCR primers

Gene	Primers (5′–3′)	Length (bp)	Annealing temperature (°C)
Recombinant plasmid AFM-1	pET-F: GATCCCGCGAAATTAATACG	1123	59.1
	pET-R: GGCCCCAAGGGGTTATGCTAG		
*blaAFM-1*	AFM-1-F: CGATTGGTGAGCAGGTGGATAAGG	336^a^	60.5
	AFM-1-R: TCGACAAGGCATTGGCGTAAGTG		
*blaAFM-1* (full length)	AFM-F: ATGATTACGAAATCGAACATCGCG	804^b^	58.0
	AFM-R: TCAGCGCAGCTTGTCGGC		

^a^Fragment amplified by specific primers for *blaAFM-1*

^b^Fragment obtained from the full-length amplification of *blaAFM-1*

### LAMP assay

The real-time fluorescence LAMP assay reaction system contained: 12.5 μL reaction solution, 1 μL Bst polymerase, 0.5 μL fluorescent dye, 3 μL ultrapure water, primer mixtures (1 μL internal primers BIP/FIP, 1 μL outer primers F3/B3, and 1 μL loop primers LF/LB at final concentrations of 1.6, 0.2, and 0.8 μM, respectively), 2 μL target DNA template, and 20 μL paraffin oil. Except for the DNA template, all other reagents were supplied by Guangzhou Bangce Biotechnology Co., Ltd. The positive control was a DNA template containing *blaAFM-1*, while the negative control was sterilized distilled water. The mixture was amplified on a LightCycler^®^ 480 Real-Time PCR System (Roche Diagnostics, Basel, Switzerland) for 60 min at 60–65 °C (optimal temperature was 63 °C). One cycle of 63 °C for 30 s at holding stage; 60 cycles of 63 °C for 15 s, 60 cycles of 63 °C for 45 s at cycling stage. The fluorescence signal value at the 45th second of each cycling cycle was collected, the fluorescence channel was used to select the FAM channel, and positive (*blaAFM-1*-positive) results generated S-shaped curves within 40 cycles.

### PCR assay

For conventional PCR, we used a 20 μL final reaction volume, including 10 μL 2 × Rapid Taq Master Mix, 7.4 μL sterilized distilled water, 0.8 μL upstream and 0.8 μL downstream AFM-1 primers (Table 2), and 1 μL DNA template. The PCR parameters were: 95 °C pre-denaturation for 5 min and then 40 cycles comprising 95 °C denaturation for 30 s, 58 °C annealing for 30 s, 72 °C extension for 30 s and 72 °C for 3 min. We electrophoresed the product on a 1.5% agarose gel, and stained and visualized it with ethidium bromide on a UV illuminator (Bio-Rad, USA). PCR amplification products were sent to Sangon Biotech for sequencing.

### LAMP assay sensitivity

Once constructed, we calculated the initial concentration of the recombinant plasmid AFM-1, DNA concentrations were calculated by the following equation (ng/μL = optical density_260_ × dilution factor × 50), then adjusted to 10^5^ copies/reaction for the LAMP assay. Copy numbers were calculated using the following formulae [copies/μL = 6.02 × 10^23^copies/mol × DNA concentration (ng/μL) × 10^−9^)/(660 × DNA length (bp)], followed by ten-fold serial dilutions to generate 10^5^, 10^4^, 10^3^, 10^2^, 10^1^, and 10^0^ copies/reaction. The minimum limit of detection was determined according to LAMP amplification curves.

The turbidity of *A. faecalis* AN70 strain bacterial solution expressing *blaAFM-1* was adjusted to 0.5 McFarland units, and diluted with 0.9% NaCl solution (Sangon Biotech). This solution was mixed with the AN70 strain suspension. This solution was then mixed with *blaAFM-1*-free sputum and *blaAFM-1*-free feces samples, respectively, to generate *blaAFM-1*-supplemented sputum simulated sample and *blaAFM-1*-supplemented fecal simulated sample, respectively. Then genomic DNA was extracted with MagBeads sputum DNA Extraction Kit (Beijing Newcomb Technology Development Co., Ltd. Beijing, China) and MagBeads Feces DNA Extraction Kit (Beijing Newcomb Technology Development Co., Ltd.), respectively. *BlaAFM-1*-free sputum and *blaAFM-1*-free feces acted as negative controls. The final simulated sample concentrations were serially diluted 10^5^, 10^4^, 10^3^, 10^2^, 10^1^, and 10^0^ copies/reaction, from high to low. The recombinant plasmid AFM-1 and simulated samples were then used in the LAMP assay to assess sensitivity. We then compared and evaluated the sensitivity of the recombinant plasmid AFM-1 and simulated samples.

### LAMP assay specificity

LAMP assay specificity was determined using 52 *blaAFM-1*-free strains (mainly collected from Nanfang hospital which is a tertiary comprehensive hospital), including six standard strains [obtained from National Center for Clinical Laboratories (Beijing, China)], 16 templates carrying the β-lactamase gene or carbapenemase gene, nine common *Enterobacterales* strains, six common non-fermenting strains, ten common Gram-positive *cocci*, four common fungi, and a human leukocyte sample. Recombinant plasmid AFM-1 was used as a positive control (Additional file 1: Table S1). Strain DNA was extracted using the Ezup column bacterial genomic DNA extraction kit (Sangon Biotech), and strains containing *blaAFM-1* were confirmed by conventional PCR and sequencing. All strains were subjected to LAMP assay, under the same conditions, with specificity identified from amplification curves.

### LAMP assay validation with clinical strains

Three hundred clinical Gram-negative carbapenem-resistant strains were collected to validate the LAMP assay. All carbapenem-resistant Gram-negative strains were identified and antimicrobial susceptibility tests were performed using the BD phoenix 100 Automated Microbiology System (BD Diagnostics, Franklin Lakes, NJ, USA). DNA from strains was extracted using the Ezup column bacterial genomic DNA extraction kit (Sangon Biotech). The blaA*FM-1* gene was detected by LAMP and conventional PCR/sequencing. LAMP and PCR reactions were processed and sequenced as previously described. Recombinant plasmid AFM-1 and sterilized distilled water were used as positive and negative controls, respectively. We then compared differences between results.

### Statistical analysis

Positive and negative numbers of the *blaAFM-1* gene detected by the LAMP assay and conventional PCR/sequencing were counted, respectively. A 2 × 2 diagnostic test characteristics of the LAMP assay against PCR/sequencing table was drawn. Sensitivity, specificity and their 95% CIs, predictive values of clinical strains were calculated by these two methods.

## Results

### Optimal LAMP primer set

**Fig. 1:**
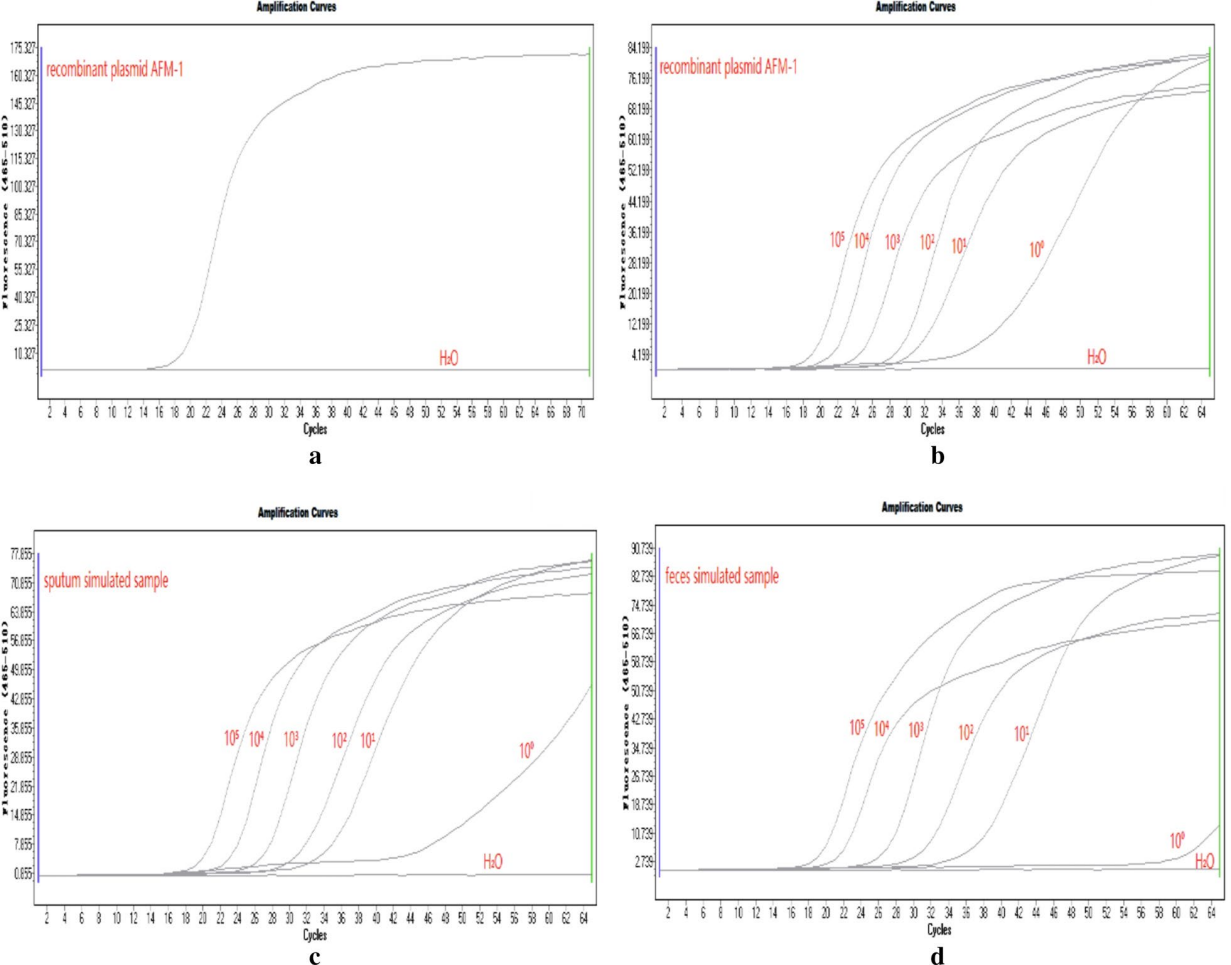
**a** Amplification curve of the optimal primer sets. **b**–**d** The sensitivity of recombinant plasmid AFM-1, a sputum-simulated sample, and a feces-simulated sample, respectively. Amplification curves were obtained by diluting recombinant plasmid AFM-1 DNA, sputum-simulated sample, and feces-simulated sample from 10^5^ copies/reaction to 10^0^ copies/reaction, respectively. Sterile distilled water was used as a negative control

Using the same reaction conditions, we screened our optimal primer sets for the highest amplification efficiency (Fig. 1a).

### LAMP assay sensitivity

LAMP amplification curves indicated that the minimum limit of detection for *blaAFM-1* from the recombinant plasmid AFM-1, two simulated (sputum simulated and feces simulated) samples were both 10^1^ copies/reaction (Fig. 1b–d).

### LAMP assay specificity

Due to LAMP specificity, except for recombinant plasmid AFM-1 which contained *blaAFM-1* and generated S-shaped curves within 40 cycles, the other 52 samples without *blaAFM-1* were not amplified. These data agreed with PCR amplification/sequencing data and indicated that the LAMP real-time assay exhibited high specificity for *blaAFM-1.*

### Clinical strain validation

**Fig. 2:**
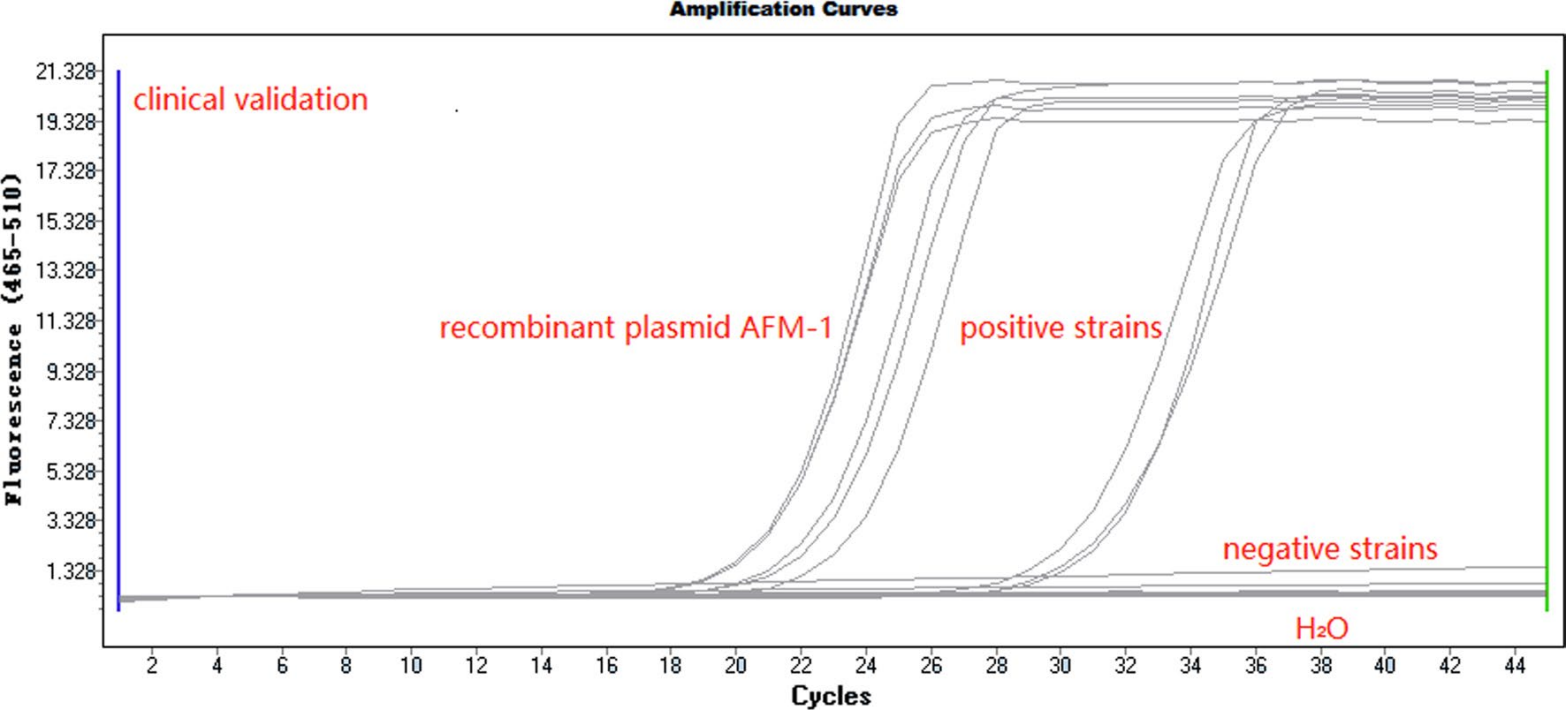
Evaluation of the LAMP method with 300 clinical strains, eight strains (out of 300) generated S-shaped type amplification curves using LAMP.

We used our LAMP assay to investigate 300 Gram-negative carbapenem-resistant strains; eight strains were identified as carrying *blaAFM-1* (Fig. 2, Additional file 1: Table S2 for the information on positive strains), in agreement with conventional PCR results. Importantly, no false positive results were identified using the LAMP assay (Table 3). For clinical validation, when compared with conventional PCR/sequencing, the sensitivity, specificity, and predictive values of the LAMP method for detecting *blaAFM-1* were 100% (Table 3). The 95% CI for sensitivity was 59.8–100%, and 98.4–100% for specificity.

**Table 3:** Assay characteristics for *blaAFM-1* detection in clinical strains (300)

	PCR/sequencing (+)*	PCR/sequencing (−)#	Total number	Sensitivity	Specificity	PPV	NPV
LAMP (+)	8	0	8	100%	100%	100%	100%
LAMP (−)	0	292	292				
Total number	8	292	300				

*The number of strains that carry the *blaAFM-1* gene; ^#^The number of strains that do not carry the *blaAFM-1* gene; PPV, positive predictive value; NPV, negative predictive value

## Discussion

MBLs hydrolyze almost all types of β-lactams (including meropenem), but not aztreonam [5, 12]. Equally, lactamase inhibitors are also ineffective against MBLs [5, 12]. Due to disease risks and limited therapies for carbapenemase-producing organisms, especially MBL-producing organisms, the rapid and early detection of carbapenemases is an urgent requirement. *BlaAFM-1*, as a new member of the subclass B1 carbapenemase gene family, was first discovered on a broad host conjugative IncW plasmid, which easily conferred *blaAFM-1* to other bacteria, therefore, AFM-1 enzyme is likely a certain risk.

To the best of our knowledge, LAMP technologies have been extensively used for bacteria, virus, and parasites [11]. LAMP internal and outer primers can increase reaction specificity, and loop primers can shorten reaction times, and increase sensitivity. It has been reported that when a recombinant plasmid containing the target gene, LAMP sensitivity was generally 100–10,000 times that of the PCR method [13]. Also, LAMP amplification does not require temperature cycling, and crude unpurified DNA can be used as a template to detect *blaAFM-1* by LAMP assay [11, 14], which greatly shortens detection times. The LAMP assay takes 1 h to complete, is user-friendly, and simply requires the mixing of reaction solution, primers, Bst polymerase and templates in a constant temperature instrument. Due to strategic LAMP primer design, these primers not only detected *blaAFM-1*, but also distinguished other MBL genes, such as *blaNDM-1*; this gene displayed the highest identity (85%) with *blaAFM-1,* but was not amplified by the LAMP assay.

Notably, given the high sensitivity of the LAMP assay, it may also produce false positive results during reaction times due to high amplification efficiencies [11, 15, 16], To reduce false positives, aerosol pollution must be prevented during procedures, therefore paraffin oil addition will prevent this [11].

This study had several limitations. Firstly, the number of clinically verified strains was small and errors may have occurred during sensitivity and specificity assay calculations, therefore larger sample sizes are required for comprehensive clinical verification. Secondly, when compared with conventional culture assays, LAMP is rapid and time-efficient. However, its application to clinical specimens remains to be thoroughly explored and verified. Lastly, the β-lactamase and carbapenemase genes, used for LAMP-specificity evaluation, were single drug resistance genes. The coexistence of two or more drug resistance genes were not evaluated for LAMP specificity, thus further investigations are needed in the future.

## Conclusions

The LAMP method was used to detect *blaAFM-1* for the first time. When compared with conventional PCR/sequencing, our method was rapid, sensitive, and specific, and is suitable for laboratory applications.

## Supplementary Information

**Additional file 1: TableS1.** Specimens for evaluating the specificity of the LAMP. **TableS2.** Information on positive strains.

## Data Availability

This article contains all the research data and materials of this research. Our data are readily available upon reasonable request.

## Abbreviations

LAMP: Loop-mediated isothermal amplification
95% CI: 95% Confidence interval
MBLs: Metallo-β-lactamases
NDM: New Delhi metallo-β-lactamase
VIM: Verona integron-encoded metallo-β-lactamase
IMP: Imipenemase metallo-β-lactamase
