# Supplementary material for: Rapid detection of a novel B1-β-lactamase gene, blaAFM-1 using a loop-mediated isothermal amplification (LAMP) assay
Source: Ann Clin Microbiol Antimicrob. 2021 Dec 7;20:80. doi: 10.1186/s12941-021-00486-z (PMC8650397; doi:10.1186/s12941-021-00486-z)
Supplement: Supplementary file 1 — Additional file 1: TableS1. Specimens for evaluating the specificity of the LAMP. TableS2. Information on positive strains. [file 12941_2021_486_MOESM1_ESM.docx]

**Additional material**

**Additional Table S1. Specimens for evaluating the specificity of the LAMP assay**

| Classification | Organism | Source | Gene | Reference | Note |
| --- | --- | --- | --- | --- | --- |
| Strains carrying *blaAFM-1* gene | Recombinant plasmid AFM-1 | / | *blaAFM-1* | This study |  |
| Standard strains | *E.coli* | ATCC25922 | / | Reference(17) | negative  strains |
|  | *Klebsiella pneumoniae* | ATCC70060 | / | Reference (17) |  |
|  | *Pseudomonas aeruginosa* | ATCC27853 | / | Reference (17) |  |
|  | *Candida albicans* | ATCC14053 | / | Reference (17) |  |
|  | *Streptococcus faecalis* | ATCC29212 | / | Reference (17) |  |
|  | *Staphylococcus aureus* | ATCC25923 | / | Reference (17) |  |
| Beta-lactamase gene-containing Strains | *Enterobacter aerogenes* | secretion | *blaNDM-1* | Reference (18) |  |
|  | *Klebsiella pneumoniae* | blood | *blaKPC-2* | Reference (17) |  |
|  | *E. cloacae* | urine | *blaIMP-4* | Reference (18) |  |
|  | *Pseudomonas aeruginosa* | sputum | *blaVIM-2* | Reference (18) |  |
|  | *E.coli* | feces | *blaSPM-1* | Reference (18) |  |
|  | *Klebsiella pneumoniae* | feces | *blaGES-1* | Clinically collected strain |  |
|  | *Acinetobacter baumannii* | urine | *blaOXA-23* | Reference (19) |  |
|  | *Acinetobacter baumannii* | feces | *blaOXA-24* | Reference (19) |  |
|  | *Acinetobacter baumannii* | feces | *blaOXA-48* | Reference (19) |  |
|  | *Acinetobacter baumannii* | feces | *blaOXA-51* | Reference (19) |  |
|  | *Acinetobacter baumannii* | sputum | *blaOXA-58* | Reference (19) |  |
|  | *E.coli* | blood | *blaTEM-1* | Clinically collected strain |  |
|  | *E.coli* | feces | *blaCMY-2* | Clinically collected strain |  |
|  | *Klebsiella pneumoniae* | feces | *blaSHV-1* | Clinically collected strain |  |
|  | *E.coli* | feces | *blaVEB-1* | Clinically collected strain |  |
|  | *Enterobacter cloacae* | sputum | *blaCTX-M-9* | Reference (20) |  |
| Common *Enterobacterales* | *Enterobacter aerogenes* | feces | / | Reference (21) |  |
|  | *E.coli* | feces | / | Reference (21) |  |
|  | *Klebsiella pneumoniae* | feces | / | Reference (21) |  |
|  | *Proteusbacillus vulgaris* | feces | / | Reference (21) |  |
|  | *Citrobacter freundii* | feces | / | Reference (21) |  |
|  | *Salmonella* | feces | / | Clinically collected strain |  |
|  | *Shigella* | feces | / | Clinically collected strain |  |
|  | *Serratia marcescens* | feces | / | Reference (21) |  |
|  | *Morganella* | feces | / | Clinically collected strain |  |
| Common Gram-positive cocci | *Enterococcus faecalis* | feces | / | Reference (22) |  |
|  | *Enterococcus faecium* | feces | / | Reference (22) |  |
|  | *Staphylococcus epidermidis* | feces | / | Reference (22) |  |
|  | *Streptococcus pneumoniae* | feces | / | Reference (22) |  |
|  | *Streptococcus agalactiae* | feces | / | Reference (22) |  |
|  | *Streptococcus aureus* | feces | / | Reference (22) |  |
|  | *Staphylococcus haemolytics* | secretion | / | Reference (22) |  |
|  | *Enterococcus avium* | feces | / | Reference (22) |  |
|  | *Enterococcus ryozoides* | feces | / | Reference (22) |  |
|  | *Staphylococcus aureus* | secretion | / | Reference (22) |  |
| Common Non-fermentative bacteria | *Pseudomonas aeruginosa* | sputum | / | Reference (21) |  |
|  | *Acinetobacter baumannii* | secretion | / | Reference (21) |  |
|  | *Pseudomonas putida* | urine | / | Reference (21) |  |
|  | *Burkholderia cepacia* | sputum | / | Reference (21) |  |
|  | *Alcaligenes faecalis* | feces | / | Reference (21) |  |
|  | *Stenotrophomonas maltophilia* | feces | / | Reference (21) |  |
| fungi | *Candida albicans* | sputum | / | Reference (22) |  |
|  | *Candida krusei* | feces | / | Clinically collected strain |  |
|  | *Candida tropicalis* | feces | / | Reference (22) |  |
|  | *Candida parapsilosis* | feces | / | Clinically collected strain |  |
|  | *Human leukocyte* | blood | / | Reference (22) |  |

**Additional Table S2. Information on strains carrying the *blaAFM-1* gene**

| strains | source | gene | GenBank ID |
| --- | --- | --- | --- |
| *Comamonas testosteroni* | feces | *blaAFM-1* | MT011984 |
| *Comamonas aquatica* | feces | *blaAFM-1* | MT180074 |
| *Stenotrophomonas maltophilia* | feces | *blaAFM-1* | CP049956 |
| *Bordetella trematum* | feces | *blaAFM-1* | CP049957 |
| *Comamonas aquatica* | feces | *blaAFM-1* | / |
| *Comamonas testosteroni* | feces | *blaAFM-1* | / |
| *Comamonas aquatica* | feces | *blaAFM-1* | / |
| *Comamonas aquatica* | sputum | *blaAFM-1* | / |
